# Prediction of the COVID disease using lung CT images by Deep Learning algorithm: DETS-optimized Resnet 101 classifier

**DOI:** 10.3389/fmed.2023.1157000

**Published:** 2023-09-07

**Authors:** J. H. Jensha Haennah, C. Seldev Christopher, G. R. Gnana King

**Affiliations:** ^1^St. Xavier’s Catholic College of Engineering, Affiliated to Anna University Chennai, Tamil Nadu, India; ^2^St. Xavier’s Catholic College of Engineering, Nagercoil, Tamil Nadu, India; ^3^Sahrdaya College of Engineering and Technology, Thrissur, Kerala, India

**Keywords:** COVID-19, transfer learning, generative adversarial network, Tuna Swarm Optimization, Resnet 101

## Abstract

As a result of the COVID-19 (coronavirus) disease due to SARS-CoV2 becoming a pandemic, it has spread over the globe. It takes time to evaluate the results of the laboratory tests because of the rising number of cases each day. Therefore, there are restrictions in terms of both therapy and findings. A clinical decision-making system with predictive algorithms is needed to alleviate the pressure on healthcare systems via Deep Learning (DL) algorithms. With the use of DL and chest scans, this research intends to determine COVID-19 patients by utilizing the Transfer Learning (TL)-based Generative Adversarial Network (Pix 2 Pix-GAN). Moreover, the COVID-19 images are then classified as either positive or negative using a Duffing Equation Tuna Swarm (DETS)-optimized Resnet 101 classifier trained on synthetic and real images from the Kaggle lung CT Covid dataset. Implementation of the proposed technique is done using MATLAB simulations. Besides, is evaluated via accuracy, precision, F1-score, recall, and AUC. Experimental findings show that the proposed prediction model identifies COVID-19 patients with 97.2% accuracy, a recall of 95.9%, and a specificity of 95.5%, which suggests the proposed predictive model can be utilized to forecast COVID-19 infection by medical specialists for clinical prediction research and can be beneficial to them.

## 1. Introduction

Lung diseases affect a significant number of individuals all over the globe. People are susceptible to a wide range of significant lung disorders, including fibrosis, asthma, pneumonia, and tuberculosis, to name just a few (1). Coronavirus infections often begin in the respiratory system, namely the lungs. Lung problems can be treated more successfully if they are discovered in the early stages (2). Image processing, Machine Learning (ML), and DL models might potentially all play a significant part in this scenario. Since December 2019, the city of Wuhan in China, along with numerous other countries, has been affected by a new coronavirus referred to as SARS-CoV-2 (3). According to Worldometer (4), as of April 18, there were 2 million and more verified patients of the virus and 150,000 and more people died.

Early detection is essential to allow the individual suspected of having the rare COVID-19 illness to be immediately isolated and to minimize infection risks to the general public as a whole because there is neither a therapeutic therapy nor a vaccine available to treat or prevent the illness (5). Chest radiography, also known as X-ray or Computed Tomography (CT) images, is a straightforward and speedy method that is used to make a diagnosis of pneumonia and COVID-19 (6). In the beginning phases of COVID-19, a ground glass pattern appears; however, it is difficult to identify this pattern near the margins of the pulmonary arteries. There have also been reports of asymmetrical, patchy, or widespread opacities of the airways associated with COVID-19. Deciphering changes in the body on such a minute scale requires a group of radiologists with extensive training (7).

Automated ways of finding such elusive abnormalities can aid with diagnosis as well as enhance early detection rates with high accuracy. Given the vast number of suspects and the limited number of radiologists who are adequately trained. DL and Artificial Intelligence (AI)-driven approaches to problem-solving have the potential to be incredibly powerful tools for addressing the problems at hand (8). AI and CT scan imaging both have the potential to provide key biomarkers for COVID-19 sickness (9). Though radiography detects sickness rapidly, several types of research have shown that employing AI and CT images to detect COVID-19-affected people is insignificant owing to a lack of sufficient images of the virus (10). Despite all of its benefits, DL has challenges at every level, from creation to application. The fundamental issue is that actual data, rather than synthetic data, is first required in most health-related systems, and as AI and DL models get more complicated, algorithms become harder to understand. In certain instances, the legal approval of the procedure is dependent on how easily the systems can be understood. There has been much written on “black-box” algorithms; in some situations, Deep Neural Networks (DNN) in particular cannot comprehend the outcome (11, 12). Even though the conclusion that was drawn from the findings of the previous investigations revealed complexities, AI and DL models aid to solve numerous disease classification systems using image processing techniques (13). Because of these reasons, this paper intends to generate synthetic images of the COVID-19 chest by using a TL method named Pix2Pix-GAN. The key contributions of this paper are outlined as follows:

- TL of the pre-trained Pix2Pix-GAN model for the public chest CT test dataset is used to predict disease.
- A novel DL approach is designed via Convolutional Neural Network (CNN) with a TSO algorithm.
- Moreover, the implemented model provides sufficient aid to the medical field and analyzes the disease severity with the help of Tuna Optimized Resnet 101.
- Based on the labels and images obtained using the openly available public Lung CT image dataset from Kaggle, create a robust prediction model that considers both COVID-19-affected and unaffected scenarios.
- The processing time is improved by cross-validating the proposed model and verifying it via a comparative study over other existing model.

The remainder of this paper describes the recent research in Section 2, while Sections 3 and 4 illustrate the problem statement and the proposed system methodologies, respectively, and Sections 5 and 6 illustrate the result, discussion, and concept of the conclusion, along with the proposed paper’s future scope.

## 2. Related works

Babukarthik et al. (14) attempt to differentiate between pneumonia due to COVID-19 and healthy lungs from CXR images (in a normal individual). A genetic DL CNN (GDCNN) and other innovative technologies were among the tools used in the research. To extract features for separating COVID-19 images from other types of images, it was trained from scratch. A new CT image retrieval approach using deep-metric learning was introduced by Zhong et al. (15). The proposed model learned the best embedding space by using multi-similarity loss, a hard-mining sampling method with an attention strategy. Training and validation of the model were carried out using a global multisite COVID-19 dataset from 3 various sources around the world. The proposed model is providing a reliable solution to evaluate CT images and manage patients for COVID-19 based on experimental findings of image retrieval and diagnostic tasks using the COVID-19 virus. COVID-19’s severity, as well as identification, were combined using multitask learning, and the CNN was suggested as a model for this purpose by Goncharov et al. (16). Findings from training a model on a large dataset were encouraging and labels were applied to a 3D chest CT by Han et al. (17). AD3D-MIL was an attention-based deep 3D multiple-instance learning method. After the potential infection area, the AD3D-MIL approach was capable of semantically building deep 3D instances. For the AD3D-MIL algorithm, Cohen’s kappa was 95.7%, Area Under Curve (AUC) was 99.99%, and overall accuracy was 97%, according to some empirical studies. Wang et al. (18) published an approach for quick COVID-19 discovery via 3D chest CT images to connect two 3D ResNets. Using the previously developed prior-attention strategy, this research created a Prior-Attention Residual Learning (PARL) model to expand residual learning. It was possible to train the model end-to-end with multi-task losses by stacking the PARL blocks. The proposed framework has been shown in experiments to considerably enhance the efficiency of screening for COVID-19. Human and machine collaboration was employed in COVID-Net, which was developed as a network designed explicitly to detect COVID-19 patients using CT scans by Wang et al. (19). Gunraj et al. (20) presented a machine-driven design examination process for building the COVIDNet-CT for determining the optimal micro and macro architecture patterns to use when building the Deep Neural Network (DNN) through automated network architectural design research. It provided more flexibility in the designing than human designing and assured the final layer met the specified operational criteria. As a result, a Deep CNN (DCNN) specifically designed for detecting COVID-19 from chest CT scans was developed using the machine-driven design exploration method. Kumar and Mahapatra (21) suggested that fractal characteristics in photographs are used for DNN construction and that lung X-ray images be used for CNN construction. The use of segmentation in conjunction with CNN architecture has been used to find the sick area (tissues) in the lung image. Jin et al. (22) provided a domain adaptation-based self-correction (DASC-Net) system for addressing the difficulties in the current COVID-19 CT images. On CT images, the developed system segmented the COVID-19-infected region. The DASC-Net system comprised of a special Attention and Feature Domain Enhanced Domain Adaptation (AFD-DA) method for addressing domain shifts with a self-correcting learning procedure designed for adaptively combining the learned network and related pseudo-labels to the transmission of associated source and target domain data for reducing overfitting to noise due to pseudo-labels. Hryniewska et al. (23) found an Explainable AI (XAI) and DL method for identifying COVID-19 via CT that depend on confounding variables. Besides, testing a DL strategy on external data was inadequate for ensuring the networks were dependent on therapeutically relevant pathophysiology since the unexpected crosscuts learned using DL frameworks can function well for datasets at new institutions, according to Geirhos et al. (24). These findings revealed that before the clinical adoption of AI systems for medical imaging, explainable DL should be taken into consideration as a need.

Yau et al. (25) examined the uses of imaging ultrasound, and their sonographic findings for COVID-19 cases compiled the most recent information and advice and discussed the possible application of POCUS in the detection, monitoring, and risk-based classification of COVID-19 cases. Xue et al. (26) created a new DL system named Dual-level Supervised Multiple Instance Learning (DSA-MIL) modules for COVID-19 patient severity rating based on Lung Ultrasound (LUS) with clinical data and exposed considerable outcomes. COVID-19 has received additional reviews for ultrasonic imaging applications. According to Saha et al. (27), the GraphCovidNet framework was a graph-isomorphic network-based framework to detect COVID-19 in chest CT images and X-rays of people with cancer. Image data were preprocessed using the GraphCovidNet model, which created an undirected graph from them and only considered their edges rather than the whole picture. The GraphCovidNet framework was capable of exactly identifying all the COVID-19 scans in a binary classification challenge. Liu and Ji (28) published a multistage Attentive TL (ATTNs) paradigm to improvise COVID-19 findings via CT scans. Moreover, the proposed model used a three-phase approach that draws on a variety of tasks and data sources. A new self-supervised learning technique that gathers semantic data from the entire lung and emphasizes the functioning of each lung area to build multiscale representations of lung CT images was developed. Finally, the method was incorporated into a TL framework so that complex patterns discovered in CT scans can be reused. The researchers found that architectures having self-attention improved efficacy more than networks without self-attention when subjected to TL. Iwendi et al. (29) addressed a Random Forest (RF) method enhanced using the AdaBoost approach to predict COVID-19. For this reason, the COVID-19 case’s geographic, as well as demographic data were utilized for the network to forecast the severity with the possibility of cure or death. The framework attained 94% accuracy and an F1 Score of 86%. Wang et al. (8) employed a GoogleNet Inception v3 CNN model to detect COVID-19.1065 CT images of people with pathogen-confirmed COVID-19 pneumonia and those who had previously been identified as having normal viral pneumonia data were gathered. To create the method, the inception TL model was changed. In 2023, Bharati et al. (30) addressed the XAI methods in healthcare. The study examined the current XAI trends and outlined the main moving directions of the field. What, why, and when of using these XAI models, as well as their effects were explained. Also, a thorough analysis of XAI approaches and a justification for how an accurate AI may be created by defining AI models for healthcare areas was provided. In 2022, Munnangi et al. (31) established a Nonlinear Cosine-based Time series Learning (NCTL) approach for COVID-19 diagnosis in India. Initially, the relevant features were selected using the nonlinear least squares regressive feature selection (NLS-RFS) model, which considers both active situations while having a lower prediction error. The cosine-based neighborhood filter technique was then used to choose the most pertinent characteristics with the shortest prediction time. Finally, the number of COVID-19 instances registered was predicted using a cosine neighborhood-based LSTM. In 2023, Podder et al. (32) developed DenseNet-169 and DenseNet-201 for Covid-19 detection by adjusting the hyperparameters via the Nadam optimization algorithm to enhance the model’s performance. 3312 CXR images were used. In 2021, Mondal et al. (33) proposed optimized InceptionResNetV2 for COVID-19 (CO-IRv2) based on the ideas of InceptionNet and ResNet with hyperparameter adjustment, global average pools, batch normalization, dense layer, and dropouts.

Table 1 summarizes the features and achievements of previous COVID-19 detection models using various AI algorithms. Several covid-prediction technologies are being developed by researchers. Although each has its drawbacks. Computational time and classification errors are major problems that still limit performance. Besides, CXR images and chest CT images were widely used in the research. Also, limited data was used in the existing research. For this reason, this paper aims to develop a novel COVID-19 disease prediction model with enhanced efficiency. Here, Lung CT images are utilized with synthetically generated data. Additionally, the performance of the classifier is enhanced by tuning the hyperparameters by optimization concepts to ensure better accuracy with minimum testing time.

**Table 1:** Features and achievements of existing COVID-19 detection systems using AI algorithms.

Authors	Methods	Features	Achievements
Babukarthik et al. (14)	GDCNN	5,000 CXR images were used Provided better performance in an unbalanced environment	Attained 98% of accuracy
Zhong et al. (15)	Deep Metric Learning-based Model	CXR images from 3 various sources were used medical histories to predict the chances of air intubation as well	Exhibited better prediction results
Goncharov et al. (16)	CNN	Chest CT images were used Identified Covid-19, pneumonia, and cancerous nodules	Revealed better results for multi-tasking including identification and severity qualification
Han et al. (17)	AD3D-MIL	Chest CT images were used Implemented a bag of instances and attention-based pooling methods to process the 3D images	Achieved 97% of accuracy and 99% of AUC
Wang et al. (18)	PARL	Chest CT images were used 2 ResNets were utilized to generate branch-wise computation	This method can be used for CAD and pulmonary nodules detection as well
Wang et al. (19)	COVID-Net	CXR images were used Provided better screening ability with explainability factors	Used large publicly available datasets with 13,975 CXR images
Gunraj et al. (20)	COVIDNet-CT	CT images were used A machine-driven design exploration method was implemented for the detection	Both COVIDNet-CT and COVIDx-CT images were utilized for computation
Kumar and Mahapatra (21)	CNN-DNN	Lung X-ray images were used Lung tissues were identified by CNN and fractal features were processed by DNN	94% of accuracy was reached
Jin et al. (22)	DASC-Net	Lung CT images were utilized AFD-DA method provided refined segmentation performance	Helped in accomplishing domain adaptation and self-correction learning in clinical research
Hryniewska (23)	XAI and DL models	Lung CT images were utilized Outlined the perspectives of radiologists and DL algorithms	Provided a checklist to meet the minimum criteria for COVID detection using DL models
Geirhos (24)	ML and DL models	Several suggestions were provided Recommendations to enhance robustness and transferability were listed	Suggested a list of approaches for real-time applications using ML and DL algorithms
Yau et al. (25)	Diagnostic Imaging Tools	POCUS images and their applicability in COVID detection were explained	The efficiency and uses of POCUS imaging in COVID-19 detection were studied
Xue et al. (26)	DSA-MIL	LUS images were used Exposed better performance in severity assessment	Accomplished with 85% of accuracy
Saha et al. (27)	GraphCovidNet	Several datasets with CXR and CT scans were used The undirected graph was used for pre-processing the images	Achieved 99% of accuracy
Liu and Ji (28)	ATTNs	Lung CT images were utilized Self-supervised learning approach was implemented	ATTNs with TL aided in attaining better performance than baseline methods
Iwendi et al. (29)	AdaBoost with RF	The quick prediction was assured History of patient data was used for computation	Attained 94% of accuracy
Wang et al. (8)	GoogleNet Inception v3 CNN	CT images were utilized Extracted the radiological features for timely detection	Achieved 89% of accuracy
Bharati et al. (30)	XAI	Present XAI trends were studied Applications of XAI in healthcare were outlined	The uses and efficiency of XAI were investigated
Munnangi et al. (31)	NCTL	Time-series data were used for the prediction time and error were minimized	Attained 97% of accuracy
Podder et al. (32)	DenseNet-169 and DenseNet-201	CXR images were utilized Exhibited computationally efficient architecture	91 and 92% of accuracy were attained by DenseNet-169 and DenseNet-201 respectively
Mondal et al. (33)	CO-IRv2	2,481 CT images were utilized Adam, Nadam, and RMSProp optimizers were implemented for COVID-19 detection	Attained 94, 96, and 96% of accuracies for Adam, Nadam, and RMSProp optimizers respectively

## 3. Problem statement

COVID-19, viral and bacterial pneumonia have recently been detected and classified using DL models mostly based on CNN. In the absence of adequate studies on scalable datasets, it remains a tough scientific topic to distinguish COVID-19-based pneumonia from viral and bacterial pneumonia. Recent ways of detecting COVID-19 and pneumonia illnesses using CT scans have several research holes, which is why this new approach was developed. Usually, CNN-related approaches fail to address the difficulties of improving picture quality. As a result, during the automated CNN feature extraction, contaminated parts of pictures were not appropriately detected as being infected at all.

In previous CNN-based studies that used the entire lung image to automatically extract features, only characteristics of affected lung areas were significant to diagnosis. To classify chest pictures, high-dimensional and irrelevant features are extracted due to the absence of ROI estimates in the images. The absence of ROI-specific traits makes it difficult to assess the degree of illness. Early lung disease diagnosis is hampered by the computationally inefficient solution provided by CNN’s lengthy training requirements. On tiny X-ray samples, COVID-19 and pneumonia detection techniques employing DL have been examined for testing, training, and validation on 10–15% of samples. Such models need a higher ratio of training to testing if they are to be considered efficient and reliable. It was necessary to train CNN models for lung disease prediction, utilizing irrelevant datasets to automatically extract feature information. A paucity of clinical data in CNN’s pre-trained networks resulted in incorrect feature extraction since COVID-19-induced pneumonia is so new. This necessitates the development of effective strategies to address the current challenges.

## 4. A novel COVID prediction scheme

The developed model provides a significant way to analyze COVID disease using the DL technique for accurate prediction. The developed model addresses the production of synthetic lung CT images, which is based on the TL-based Pix2Pix GAN.

### 4.1. Dataset

**Figure 1:**
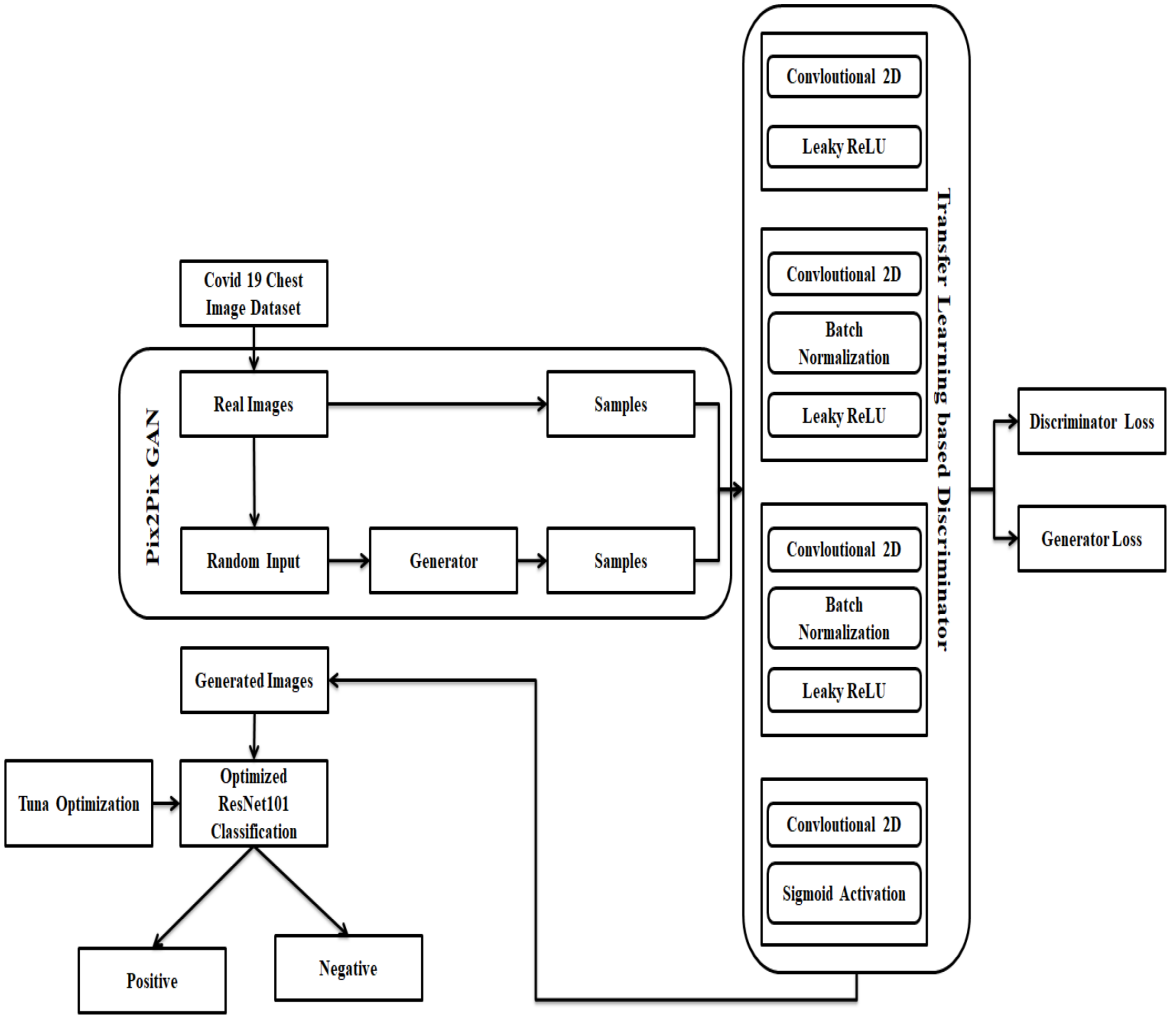
Architecture of the suggested COVID prediction model.

The dataset for the COVID-19 photos was found on several different platforms. In addition, a collection of lung-imaging CT scans is used and utilized data from the Kaggle repository for testing and validation purposes in the link SARS-COV-2 Ct-Scan Dataset | Kaggle [Access Date 08-12-2022). There are two types of data in this set. Data on people infected with COVID-19 is presented first, followed by data on people who aren’t infected with COVID-19, or “normal” people. Classification and identification of COVID-19 infection are all possible uses for this information. There are 2,482 photos in the dataset, 1,252 of which are of patients with COVID-19, and 1,230 of which are not. From these real images, the pix 2 pix GAN is used to generate synthetic data which employs the data augmentation process to eliminate the overfitting issues in Resnet 101 classifier. Here, the images are loaded, rescaled, and converted to grayscale format by variating the Loss function L1. Now, the augmented dataset consists of 7,446 images, The process of pix 2 pix GAN in this research is explained in Section 4.2. Figure 1 portrays the architecture of the suggested COVID-19 prediction model using the DL algorithm.

### 4.2. Transfer learning-based pix 2 pix GAN

The corona disease prediction process is considered as a mapping of input corona data Z to its matching illness prediction data $\hat{Z}\;$ through a mapping function d as $d\left(Z\right)=\hat{Z}$ and it is non-invertible in the ideal case, meaning that the original image cannot be recovered from the de-identification image. To produce images with a specified pattern of distribution, researchers often use GANs. This study’s goal is to generate chest images by meeting a series of requirements; therefore, a GAN-based architecture seems like an obvious choice for learning the mapping function d . Generating input data is the first step of GAN, and it is followed by a discriminator network that determines whether or not an input image belongs to the original data distribution or one learned by the generator network; this is the simplest kind of GAN to understand and implement (i.e., fake data). To achieve both the identity code and its phases using this GAN’s fundamental structure, however, is not conceivable. Conditional GANs seem to be the best solution if a collection of secondary features is used for input data distribution learning. Loss functions for each auxiliary piece of information are generated via conventional metrics/Neural Networks (NNs). Also, the aggregate loss of each of the secondary models and metrics is collectively reduced with the loss function for the discriminator.

As a starting point, build the Optimized ResNet 101 using two independent deep networks, a pix-2-pix GAN, and TL, and design loss functions. There must be no traces of the input image in the produced image, and it must also retain the qualities of that data to be considered accurate. In Figure 1, a generator and a discriminator are combined to form one unit. The generator creates an image. Also, the discriminator returns a score (GAN loss) showing whether the created image falls inside the gallery set’s probability distribution. Both contrastive and cross-entropy loss are calculated by the verifier networks to reflect how similar or different an image produced from an input image is from the original image. Losses from a specific epoch are sent back to build a better corona image based on the weighted combination of these data later. The combination is more appropriate for the various constraints or situations.

Reconstruction of the under-sampled chest CT data was achieved via TL using a pre-trained Pix2Pix-GAN model (34). Repurposing an already-trained model for another task is known as TL. To build a model from scratch for uncommon or developing illnesses, TL is most beneficial when there are not enough training examples available. Using TL, the model parameters are already excellent and just require a few minor tweaks that are best suited to the new functions at hand, making the process much easier. It is possible to employ a pre-trained network for new tasks in one of two ways. When a pre-trained model is used to extract features instead of being used to classify, then there is no change in the pre-trained model’s internal weights to fit the new tasks. Another option is to fine-tune the whole network, or a part of it, for the new purpose. A trained model is used as a starting point and modified throughout training. With the help of the GAN-based TL technique, the suggested model can combine domain adaptation and feature learning in one training phase. The generator $F_{d}$ ( ⋅ ) and the discriminator $F_{s}$ ( ⋅ ) are the only two components of a GAN model that are straightforward. When a multi-layer mapping function is used to generate the feature vectors, the raw sampling points z are used as the source domain, and $\theta_{d}$ be defined as $\;O_{d}=F_{s}\left(d,\theta_{s}\right)$ , where *O_d_* is the probability that d comes from the target rather than the source domain and s is the discriminator parameter. While in training mode, the discriminator tries to optimize $O_{d}$ such that the produced feature $d_{r}$ from a target, a domain can be discriminated from the generated feature d from the source. Contrary to this, the generator’s goal is not to increase the $O_{d}$ , but rather to deceive the discriminator by generating bogus samples with a distribution that closely resembles that of the original domain. The generator’s strategy function S is expressed as given in Equation (1) are captured by the GAN model as the minimax model progresses.


(1) $$C\left(S,F\right)=W_{z^{a}\sim {\bar{Z}}^{a}}\left[\log\;F_{s}\left(z^{a}\right)\right]+W_{z^{r}\sim {\bar{Z}}^{r}}\left[\log\left(1-F_{s}\left(S\left(z^{r}\right)\;\right)\right)\right]$$

Equation (1) can be rewritten as stated in Equation (2),


(2) $$\begin{array}{l} \underset{S}{\min}\underset{F}{\max}C\left(S,F\right)=\\ W_{z^{a}\sim O_{s}\left(z\right)}\left[\log S\left(\bar{z}\right)\right]+W_{z^{a}\sim O_{s}\left(z\right)}\left[\log\left(1-S\left(\bar{z}\right)\right)\right] \end{array}$$

For the first time in the history of corona disease prediction, this paper concentrates on creating more realistic images with unique morphological traits and obvious borders. To help with the model training, this discriminator’s last layer calculates the loss function. Using the noise distribution provided by O_m_, the generator fed using an arbitrary noise vector (assume m ). $O_{\mathrm{data}}$ is likewise assumed to be a distribution of input data and $O_{\mathrm{gen}}$ is a distribution of output data learned via a generator at a given iteration Using the training sample, the generator produces a mapping $F:\left(Z,m\right)\mapsto \hat{Z}$ , in which $Z\in O_{\mathrm{data}}$ and $\hat{Z}\in$ $O_{\mathrm{gen}}$ . Multiple epochs are used to train the GAN, and the training procedure is stopped once the 2 distributions $O_{\mathrm{gen}}$ as well as $O_{\mathrm{data}}$ are comparable.

The value function C is used by the discriminator and generator in a min-max process (S,F) . To minimize its reward C(S,F) , the discriminator tries to diminish its reward, while the generator tries to maximize its loss. To develop high-resolution images with synthetic images, the generator must be close to the deeper network. More convolution layers and time spent training for up-sampling are required for a deeper network. Initially, input the original image data and scaled it to 128∗128∗3 before using the GPU for training. To match the generator’s pixel values, the image was resized to [1,1] . The tanh activation function is why it was issued. Fake samples are generated by feeding a 100∗1 noise vector into the generating network. High-quality synthetic images were created using four convolution layers and ReLU activation. It is the generator’s goal to decrease the next loss function, whereas the discriminator’s goal is to increase it. With the original input data x and random noise variable $O_{ebc}\left(ebc\right)\;$ added, the generator creates samples F.(ebc) . Over the data distribution $O_{s}$ , S(Z) is the discriminator’s estimate of how likely it is that the actual instance z is real. The discriminator’s estimate of the likelihood that a false instance is genuine is S(F(ebc)) . To deceive the discriminator, the generator attempts to produce almost flawless images. Contrast this with an algorithm that attempts in vain to discriminate between actual and false data samples until the generator’s created data cannot be separated from real data samples.

To accurately categorize a given image into one of two categories: genuine or false from the distributions $O_{\mathrm{data}}$ and $O_{\mathrm{gen}}$ correspond to the two classes. Assume the ground truth labels for $O_{\mathrm{data}}$ and $O_{\mathrm{gen}}$ are [0,1] and [1,0] respectively. A generator that has completed its training phase can generate pictures that are quite close to the source data in terms of appearance. A CNN with four layers serves as the discriminator model. The activation function of the Leaky ReLU is employed in the first and second convolutional layers of this model. The generator encoder block’s network topology is carried through to the third to sixth levels. Finally, a convolutional layer with a stride of 1 is applied as the last step. The encoder’s primary goal is to learn the representation feature, whereas the discriminator’s primary goal is to identify the discriminating feature as shown in Equation (3).


(3) $$K_{\mathrm{recons}}^{\mathrm{pixels}}=W_{p\sim S_{\mathrm{encoder}}\left(Z\right),Z\sim U_{\mathrm{real}}}\left[\left||\kappa \left(p\right)-\tau \left(Z\right)|\right|\right]$$

where the discriminator’s feature map is p , the κ function processes p , and the decoder’s function r reflects processing on sample Z from real images $U_{\mathrm{real}}$ . The rectangular portion of the original picture is first resized. A picture feature map is extracted from the major component of the discriminator, and the decoder can build a good reconstruction from this data Here, an adversarial loss for GAN is proposed, taking into account both real and false samples that fall inside the margins. In the generator training step, artificial samples outside of the bounds that include misleading local patterns are disregarded as demonstrated in Equation (4).


(4) $$\begin{array}{l} K_{S}=-W_{z\sim U_{\mathrm{real}}}\left[\min\left(0,-1+S\left(Z\right)\right)\right]-W_{m\sim o\left(m\right)}\\ \;\;\;\;\;\;\;\;\;\;\;\;\;\;\;\;\;\;\;\;\;\;\;\;\;\;\left[\min\left(0,-1-S\left(F\left(m\right)\right)\right)\right]+K_{\mathrm{recons}}^{\mathrm{pixels}} \end{array}$$

(5) $$K_{S}=-W_{m\sim o\left(m\right)}\left[S\left(F\left(m\right)\right)\right]$$

The $u^{th}$ training pattern is called $t_{u}$ which represents the input picture for the discriminator at this specific epoch. If $t_{u}$ falls inside $O_{\mathrm{data}}$ or $O_{\mathrm{gen}}$ ’s probability distribution $t_{u}$ ∈ $O_{\mathrm{data}}$ or $t_{u}$ ∈ $O_{\mathrm{gen}}\;$ , the discriminator differentiates between the two. The generator-discriminator network’s mini-max loss function can be expressed as stated in Equation (6).


(6) $$\begin{array}{l} K_{\mathrm{patchGAN}}\left(\left\{t_{1},t_{2},\ldots t_{B}\right\},S\left(Z,t\right)\right)=\\ W_{Z,\hat{Z}\in Odata\left(Z,\hat{Z}\right)}\left[\log\left(S\left(Z,\hat{Z}\right)\right)\right]+\\ W_{Z\in Odata\left(Z,\hat{Z}\right),m\in O_{m}\left(m\right)}\left[\log\left(1-S\left(Z,F\left(Z,m\right)\right)\right)\right] \end{array}$$

In this case, W stands for the expectation operator. Also, training the GAN using the loss function of Equation (6) failed to adequately retain the structure. Rendering images might seem precise. Adding a structural similarity loss term ( $K_{\mathrm{ssim}}\left(Z,\hat{Z}\right)$ ), together with the loss function, allows us to maintain the non-biometric characteristics mentioned above while still generating an appealing corona disease prediction picture at high resolution. The similarity, in contrast, brightness, and structure between Z and $\hat{Z}$ is used to calculate this loss term, which is a composite measure of all three. The Structural Similarity Index (SSIM) among 2 input pictures Z and $\hat{Z}$ is formally stated as portrayed in Equation (7).


(7) $$SSIM\left(Z,\hat{Z}\right)=k{\left(Z,\hat{Z}\right)}^{\alpha }\cdot x{\left(Z,\hat{Z}\right)}^{\beta }\cdot a{\left(Z,\hat{Z}\right)}^{\gamma }$$

where, the parameters k,x, and a are defined as given in Equations (8)–(10) respectively.


(8) $$\;k\left(Z,\hat{Z}\right)=\frac{2\mu_{Z}\mu_{\hat{Z\;}}+x_{1}}{\mu_{Z}^{2}+\mu_{\hat{Z}}^{2}+x_{1}},$$

(9) $$x\left(Z,\hat{Z}\right)=\frac{2\sigma_{Z}\sigma_{\hat{Z\;}}+x_{2}}{\sigma_{Z}^{2}+\sigma_{\hat{Z}}^{2}+x_{2}},$$

(10) $$a\left(z,\hat{z}\right)=\frac{\sigma_{Z,\hat{Z}}+x_{3}}{\sigma_{Z}\sigma_{\hat{Z}}+x_{3}}$$

Hither, $\mu_{Z}$ and $\mu_{\hat{Z}}$ indicate the input’s Z and $\hat{Z}$ mean intensities, $\sigma_{\hat{z}}^{2}$ signifies the variances of intensities of Z and $\hat{Z}$ and $\sigma_{Z},\sigma_{\hat{Z\;}}$ indicates Z and $\hat{Z}$ covariance. Constants $x_{1}$ , $x_{2}$ and $x_{3}$ are defined as expressed in Equations (11)–(13) respectively.


(11) $$x_{1}={\left(j_{1}K\right)}^{2}$$

(12) $$x_{2}={\left(j_{2}K\right)}^{2}$$

(13) $$x_{3}=\frac{x_{2}}{2},$$

Here, $j_{1}=0.01,K$ points to pixel values’ dynamic range. Besides, Equation (14) is used to calculate $K_{\mathrm{ssim}}\left(z,\hat{z}\right)$ .


(14) $$K_{ssim}\left(z,\hat{z}\right)=\frac{1}{2}\left(1-SSIM\left(z,\hat{z}\right)\right)$$

If $k_{XGAN}$ portrays generators and discriminator’s loss function to be minimized without applying any condition, then $k_{XGAN}$ is stated as specified in Equation (15).


(15) $$k_{\mathrm{XGAN}}\left(Z,\hat{Z},S\left(Z,\hat{Z}\right)\right)=k_{\mathrm{patchGAN}\left(Z,\hat{Z}\right)}+\Phi K_{ssim}\left(Z,\hat{Z}\right)$$

where Φ indicates a positive constant in the interval [0,1] . The value must be carefully set for attaining a better balance between successful de-identification and preservation of structural similarities. The ideal equilibrium is achieved at 0.25 to acquire accurate images.

### 4.3. Optimized Resnet 101 classification

Here, the classifier parameter is tuned using the improved Tuna Swarm Optimization (TSO) method (35), which is based on the tuna’s foraging behavior and offers excellent global searchability. Tuna has a streamlined physique in tropical and subtropical warm seas because they are marine fish. After seeing the target, the tuna swarm circles the prey in a bionic manner, searching for prey in groups of individuals and populations that are randomly swimming in the water. Finally, the tuna will progressively shrink the surrounding range, which is dominated by spiral and parabolic patterns. Swim rapidly to grab prey like mackerel after you have found a good spot.

Initialization: Here, tuna is arbitrarily dispersed concerning their current position as given in Equation (16).


(16) $$O_{u}^{0}=rand\cdot \left(K_{\mathrm{up}}-K_{\mathrm{low}}\right)+K_{\mathrm{low}},u=1,2,\ldots ,N_{\mathrm{pop}}$$

in which $O_{u}^{0}\;$ specifies the initial location of the $u^{th}$ tuna, $K_{\mathrm{up}}$ and $K_{\mathrm{low}}$ points to search range of tuna, $N_{\mathrm{pop}}$ refers to tuna fish population count, and rand signifies an arbitrary vector uniformly distributed among (0,1) .

1. Spiral Foraging: When immature fish swarms confront predators, the fish school creates a thick shape, continually altering its swimming direction, making it impossible for the predator to secure its location as defined in Equation (17).

(17) $$O_{u}^{r+1}=\{\begin{array}{c} x_{1}\cdot \left(O_{\mathrm{best}}^{r}+\lambda \cdot \left|O_{\mathrm{best}}^{r}-O_{u}^{r}\right|\right)+x_{2}\cdot \;O_{u,u=1}^{r}\\ \begin{array}{l} x_{1}\cdot \left(O_{\mathrm{best}}^{r}+\lambda \cdot \left|O_{\mathrm{best}}^{r}-O_{u}^{r}\right|\right)+x_{2}\cdot \;O_{u-1}^{r},\\ u=2,3,\ldots ,N_{\mathrm{pop}} \end{array} \end{array}$$

1. The tuna swam in a helical formation, encircling and engulfing their victim. The tuna can change places at any moment and pursue the prey. A meta-heuristic algorithm like the tuna swarm performs a global search in the early stages before progressively shifting its focus to a local search as described in Equations (18)–(22).

(18) $$O_{u}^{r+1}=\{\begin{array}{c} x_{1}\cdot \left(O_{\mathrm{rand}}^{r}+\lambda \cdot \left|O_{\mathrm{rand}}^{r}-O_{u}^{r}\right|\right)+x_{2}\cdot \;O_{u}^{r},u=1\\ \begin{array}{l} x_{1}\cdot \left(O_{\mathrm{rand}}^{r}+\lambda \cdot \left|O_{\mathrm{rand}}^{r}-O_{u}^{r}\right|\right)+x_{2}\cdot \;O_{u-1}^{r},\\ u=2,3,\ldots ,N_{\mathrm{pop}} \end{array} \end{array}$$

1. The spiral foraging procedure is stated in Equation (23).

(19) $$x_{1}=l+\left(1-l\right).\frac{r}{R}$$

(20) $$x_{2}=\left(1-l\right)+\left(1-l\right)\cdot \frac{r}{R}$$

(21) $$\lambda =e^{mv}\cdot \cos\left(2\pi v\right)$$

(22) $$m=\exp\left(3\cos\left(\left(\frac{R-r+1}{r}\right)\pi \right)\right)$$

(23) $$O_{u}^{r+1}=\{\begin{array}{c} ,x_{1}\cdot \left(O_{r\;\mathrm{and}}^{r}+\lambda \cdot \left|O_{\mathrm{rand}}^{r}-O_{u}^{r}\right|\right)+x_{2}\cdot O_{u-1}^{r},\;u=2,3,\ldots ,Bx_{1}\cdot \left(O_{\mathrm{rand}}^{r}+\lambda \cdot \left|O_{\mathrm{rand}}^{r}-O_{u}^{r}\right|\right)+x_{2}\cdot O_{u}^{r},\;u=1\;\;if\;rand<\frac{r}{R}\\ ,\;x_{1}\cdot \left(O_{\mathrm{best}}^{r}+\lambda \cdot \left|O_{\mathrm{best}}^{r}-O_{u}^{r}\right|\right)+x_{2}\cdot O_{u-1}^{r},\;u=2,3,\ldots ,Bx_{1}\cdot \left(O_{\mathrm{best}}^{r}+\lambda \cdot \left|O_{\mathrm{best}}^{r}-O_{u}^{r}\right|\right)+x_{2}\cdot O_{u}^{r},\;u=1\;if\;rand>\frac{r}{R} \end{array}$$

1. Parabolic Foraging: At times, tuna would hunt their food in the parabolic shape, using a school of larval fish as their point of reference. It is common for one section of the swarm to adopt a spiral shape, while the other part adopts a parabolic shape. The mathematical model of the parabolic shape assumes that the chance of selecting the two hunted and released is 50% as expressed in Equations (24), (25).

(24) $$O_{u}^{r+1}=\{\begin{array}{c} \begin{array}{l} O_{\mathrm{best}}^{r}+rand\cdot \left(O_{\mathrm{best}}^{r}-O_{u}^{r}\right)+\\ g\cdot j^{2}\cdot \left(O_{\mathrm{best}}^{r}-O_{u}^{r}\right),\mathrm{if}\;rand<0.5 \end{array}\\ g\cdot j^{2}\cdot O_{u}^{r},\;\mathrm{if}\;rand>0.5 \end{array}$$

(25) $$j={\left(1-\frac{r}{R}\right)}^{\frac{r}{R}}$$

R represents the Iteration, $x_{1}$ , and $x_{2}\;$ are the weight control parameters; l refers to the constant, v is the arbitrary number among (0,1) , J signifies the spiral equation; m denotes the spiral equation parameter, r is the current iteration number. There are four possible positions for the $O_{\mathrm{best}}^{r}$ iteration of the population: random, O _(rand)_, and optimum. O _(rand)_ represents the $O_{\mathrm{best}}^{r}\;$ iteration of the population’s random location, while O _best_ represents the ideal position for the population’s $r^{th}$ iteration. A random integer between zero and one between r and R is used to determine how many times to repeat the process (0,1).

Generally, the traditional TSO is efficient in solving complex optimization problems and engineering problems. However, the traditional TSO performance is limited due to complexities in deciding the behavior from an individual tuna’s perspective. Often tends to premature convergence and local optima issues due to the neighborhood search pattern of tuna. Intending to solve these issues, the traditional update expression given in Equation (18) is replaced by the Duffing equation concepts. Since Duffing equation is better at solving nonlinear and chaotic problems, the concepts are used to update the tuna’s position update to evade the local optimal and low convergence issues as shown in Equation (26).


(26) $$O_{u}^{r+1}=\{\begin{array}{c} x^{\prime \prime }+\rho x^{\prime }+\tau x+\varphi x^{3}=ℶ\cos\left(\vartheta T\right),\;if\;rand<0.5\\ y^{\prime \prime }+2\hat{\rho }y^{\prime }+y+\hat{\varphi }y^{3}=\cos\left(\delta T\right),\;\;if\;rand>0.5 \end{array}$$

Here, x indicates the tuna ifrand<0.5 at iteration T , $x^{\prime }$ denotes the first order of x , $x^{\prime \prime }$ points to the second order derivative of x , the constants used in Equation (26) have the default values as ρ=0.02 , τ=1 , φ=5 , ℶ=8 , and ϑ=0.5 . moreover, y indicates the tuna ifrand>0.5 at iteration T , $y^{\prime }$ denotes the first order of y , $y^{\prime \prime }$ points to the second-order derivative of y , the variables $\left(\hat{\rho },\hat{\varphi },\delta \right)$ used in Equation (26) are estimated based on Equations (27)–(29) respectively.


(27) $$\hat{\rho }=\frac{\rho }{2\sqrt{\tau }}$$

(28) $$\hat{\varphi }=\frac{\varphi {ℶ}^{2}}{\tau^{3}v}$$

(29) $$\delta =\frac{\vartheta }{\sqrt{\tau }}$$

Now, the key parameters of the Resnet 101 classifier are optimized with the improved DETS optimizer. The 1st hidden layer nodes count $B_{1}$ , the 2nd hidden layer nodes count $B_{2}$ , the iterations count $B_{\mathrm{iter}}$ , and the learning rate $K_{E}$ all had a significant influence on classification outcomes in the Resnet 101 network (36). The DETS optimizer is used to optimize key variables $\left\{B_{1},\;B_{2},\;B_{\mathrm{iter}},\;K_{E}\right\}\;$ of the Resnet 101 network. Nodes in one layer are linked to nodes in the next, but nodes inside a layer are not connected at all. When the unit vector of the visible layer $c=\left(c_{1},\;c_{2},\ldots ,\;c_{n}\right)$ and hidden layer $g=\left(g_{1},\;g_{2},\ldots ,\;g_{b}\right)$ is noted, the joint state energy function among every neuron’s pair of visible and hidden layers of Resnet 101 W(c,g) can be expressed as stated in Equation (30).


(30) $$W\left(\theta \right)=-\overset{b}{\underset{h=1}{\sum }}\overset{n}{\underset{u=1}{\sum }}q_{uh}c_{u}g_{h}-\overset{n}{\underset{u=1}{\sum }}l_{u}c_{u}-\overset{b}{\underset{h=1}{\sum }}v_{h}g_{h}$$

Where, $l_{u}\;$ indicates visible layer bias, $v_{h}\;$ refers to hidden layer bias, $q_{uh}$ denotes the weight matrix to connect visible and hidden layers, and $\theta =\left\{l_{u},q_{uh},v_{h}\right\}\;$ portrays Resnet 101 network’s parameter. The distribution function of the visible and concealed components can be calculated using the energy function as shown in Equations (31) and (32).


(31) $$O\left(\theta \right)=\frac{1}{M_{\theta }}e^{-W\left(\theta \right)}$$

(32) $$M_{\theta }=\underset{c}{\sum }\underset{g}{\sum }e^{-W\left(\theta \right)}$$

where $M_{\theta }$ is a normalizing partition function.

The following logical function is used to represent conditional probability in Resnet 101, which is derived from the Bayesian formula’s premise as portrayed in Equations (33)–(35).


(33) $$O\left(g\right)=\sigma \left(l_{u}+\overset{b}{\underset{h=1}{\sum }}q_{uh}g_{h}\right)$$

(34) $$O\left(c\right)=\sigma \left(v_{h}+\overset{n}{\underset{u=1}{\sum }}q_{uh}c_{u}\right)$$

(35) $$\sigma \left(z\right)=\frac{1}{1+e^{-z}}$$

in which σ point to the Logistic function.

The first step is to initialize the layer which is visible to the user. The hidden neuron’s conditional probability is then determined using the visible layer’s value and the conditional probability equation. The guidelines for updating relevant parameters as a result of repeatedly doing this procedure are demonstrated in Equations (36)–(38).


(36) $$\Delta q_{uh}=\varepsilon \left({\langle c_{u}g_{h}\rangle }_{\mathrm{data}}-{\langle c_{u}g_{h}\rangle }_{\mathrm{recon}}\right)$$

(37) $$\Delta l_{u}=\varepsilon \left({\langle c_{u}\rangle }_{\mathrm{data}}-{\langle c_{u}\rangle }_{\mathrm{recon}}\right)$$

(38) $$\Delta v_{h}=\varepsilon \left({\langle g_{h}\rangle }_{\mathrm{data}}-{\langle g_{h}\rangle }_{\mathrm{recon}}\right)$$

where 𝜀 is the feature learning rate and ${\langle \cdot \rangle }_{\mathrm{data}}{\langle \cdot \rangle }_{\mathrm{recon}}\;$ represent the model is a representation of the data, and the model’s anticipated values reflect the data. Using this criterion, an appropriate weight is found; the same process can be followed until all Resnet 101 weights have been adjusted using this criterion. The following are the steps taken by the DETS optimizer to improve the parameter calculations for Resnet 101.

Step 1: After preprocessing, dimensionality reduction, and normalization, the actual data are imported and separated into training as well as testing samples.

Step 2: The Resnet 101 network topology was created by the sample data requirements.

Step 3: After the settings of the Resnet 101 were initialized, as well as the DETS optimizer parameters, comprising the tuna population $\;\left\{B_{1},\;B_{2},\;B_{\mathrm{iter}},\;K_{E}\right\}$ and S samples are set.

Step 4: As the tuna fish continued to operate, the fitness value and the position were updated in real-time using formulae (30), (31), and (32); within the iteration range. If the new site was superior, the old one would have been demolished and rebuilt.

Step 5: The value of the appropriate combination of Resnet 101 parameters $\;\left\{B_{1},\;B_{2},\;B_{\mathrm{iter}},\;K_{E}\right\}$ was stored based on the optimum fitness value.

Step 6: Resnet 101 was trained and the appropriate parameters were stored based on the ideal parameter combination $\;\left\{B_{1},\;B_{2},\;B_{\mathrm{iter}},\;K_{E}\right\}$ .

**Figure 2:**
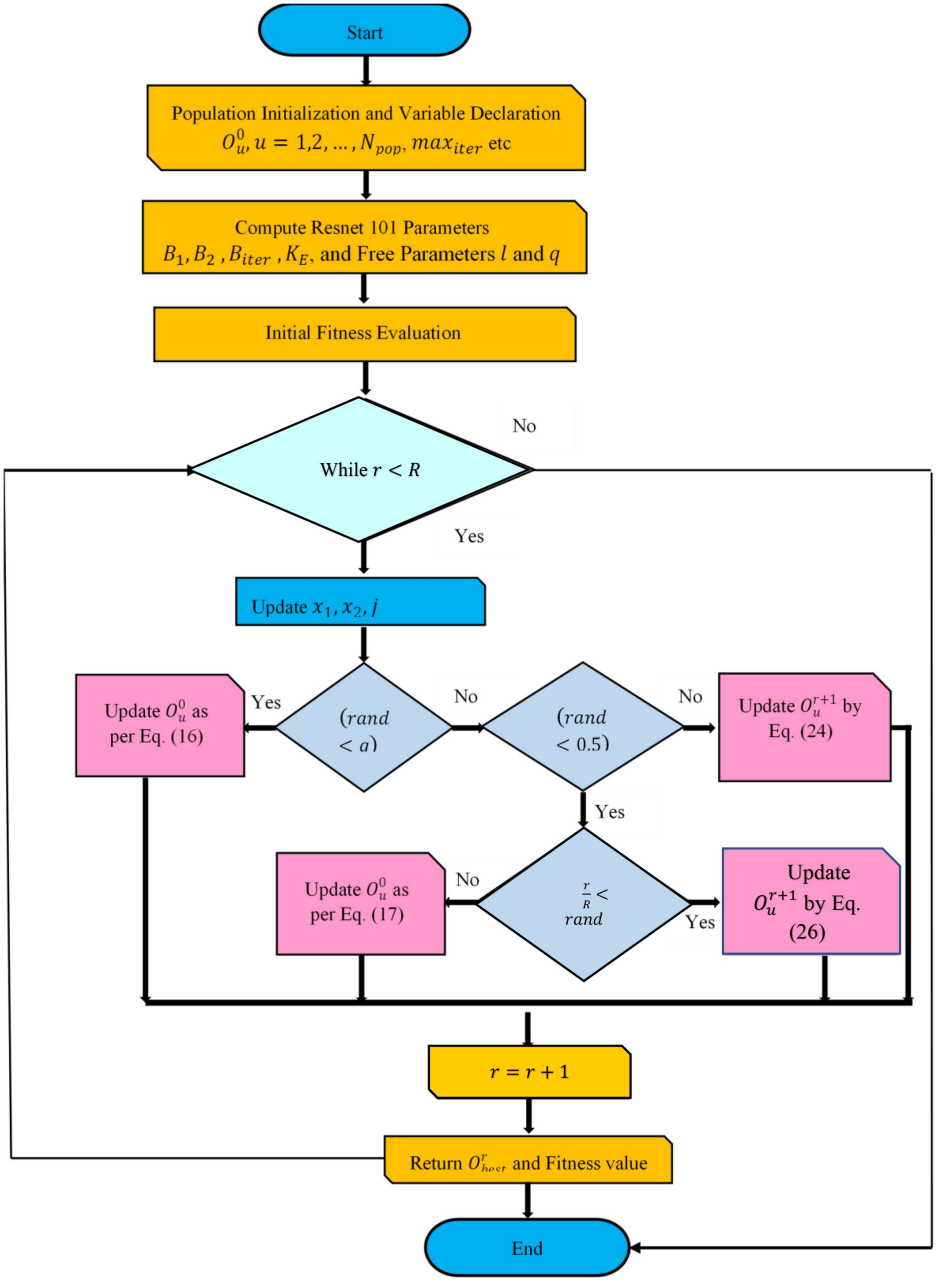
Flowchart of proposed optimized Resnet 101 using DETS optimizer.

Because of step 6, Resnet 101 was able to generate the categorization results. Finally, the covid positive and non-covid images can be classified. Figure 2 depicts the flowchart of the proposed model. Algorithm 1 and 2 shows the pseudocode of the DETS optimizer and the proposed optimized Resnet 101 model.

#### Algorithm 1: Pseudocode of Proposed DETS Optimizer.

**Input:** $N_{pop}$ **and** $max_{iter.}$

**Output: Optimal** $O_{best}^{r}$

Declare initial population $O_{u}^{0},u=1,2,\ldots ,N_{pop}$

Free parameters l, and q , where q=0.05

While (r<R)

Fitness estimation

Update $O_{best}^{r}$

For every u do

Update $x_{1},x_{2},j$

If (rand<q) then

Update $O_{u}^{0}$ as per Eq. (16)

Else if (rand≥q) then

If (rand<0.5) then

If $\left(\frac{r}{R}<rand\right)$ then

**Update** $O_{u}^{r+1}$ **by** **Eq.** (18)

If $\left(\frac{T}{max_{iter}}\geq rand\right)$ then

Update $O_{u}^{r+1}$ by Eq. (17)

Else if (rand≥0.5) then

Update $O_{u}^{r+1}$ as per Eq. (26)

End for

r=r+1

End while

Return $O_{best}^{r}$ and fitness value

End

#### Algorithm 2: Pseudocode of Optimized Resnet 101

**Input: Generated image**

**Output: Classified output**

for char ∈ n do and char ← binary image char (char)

har←packing(char);attach(N,char)

end for

$barlength\leftarrow \frac{B}{7}$

N←Bar(N.TO)

j←RandomTO([1,b−1])

$X_{1}\left(z_{1},t_{1}\right)\leftarrow TO$

$X_{1}\left(z_{1},t_{1}\right)\leftarrow N+TO$

for char∈N do

optimization $\leftarrow char\;z^{2}$

end for

return $\left(\left\{B_{1},B_{2},B_{iter},K_{E}\right\}\right)$

n←split(n)

n=data

attach (tuning parameter)

$\Delta q_{uh}=\varepsilon \left({\langle c_{u}g_{h}\rangle }_{data}-{\langle c_{u}g_{h}\rangle }_{recon}\right)$

$\Delta l_{u}=\varepsilon \left({\langle c_{u}\rangle }_{data}-{\langle c_{u}\rangle }_{recon}\right)$

$\Delta v_{h}=\varepsilon \left({\langle g_{h}\rangle }_{data}-{\langle g_{h}\rangle }_{recon}\right)$

end

## 5. Experimental setup

The presented COVID-19 disease diagnosis approach via the optimized ResNet model was implemented in MATLAB on Intel core^®^ core i3 processor 7,020 U@2.3 GHz, 8 GB RAM, 64-bit operating system. Lung CT images are used for implementation. For experimentation, the proposed scheme employed a holdout of 70:30 training-to-testing data ratio of Optimized Resnet 101 to categorize covid-19 photos. The efficacy of the proposed COVID-19 detection scheme is achieved using simulation results. Here, the existing models like Fractal Features-based Deep Neural Network (FFDNN) (21), CCN (21), Resnet 101 (36), and traditional TSO-based Resnet are compared with the proposed model

### 5.1. Performance analysis

**Figure 3:**
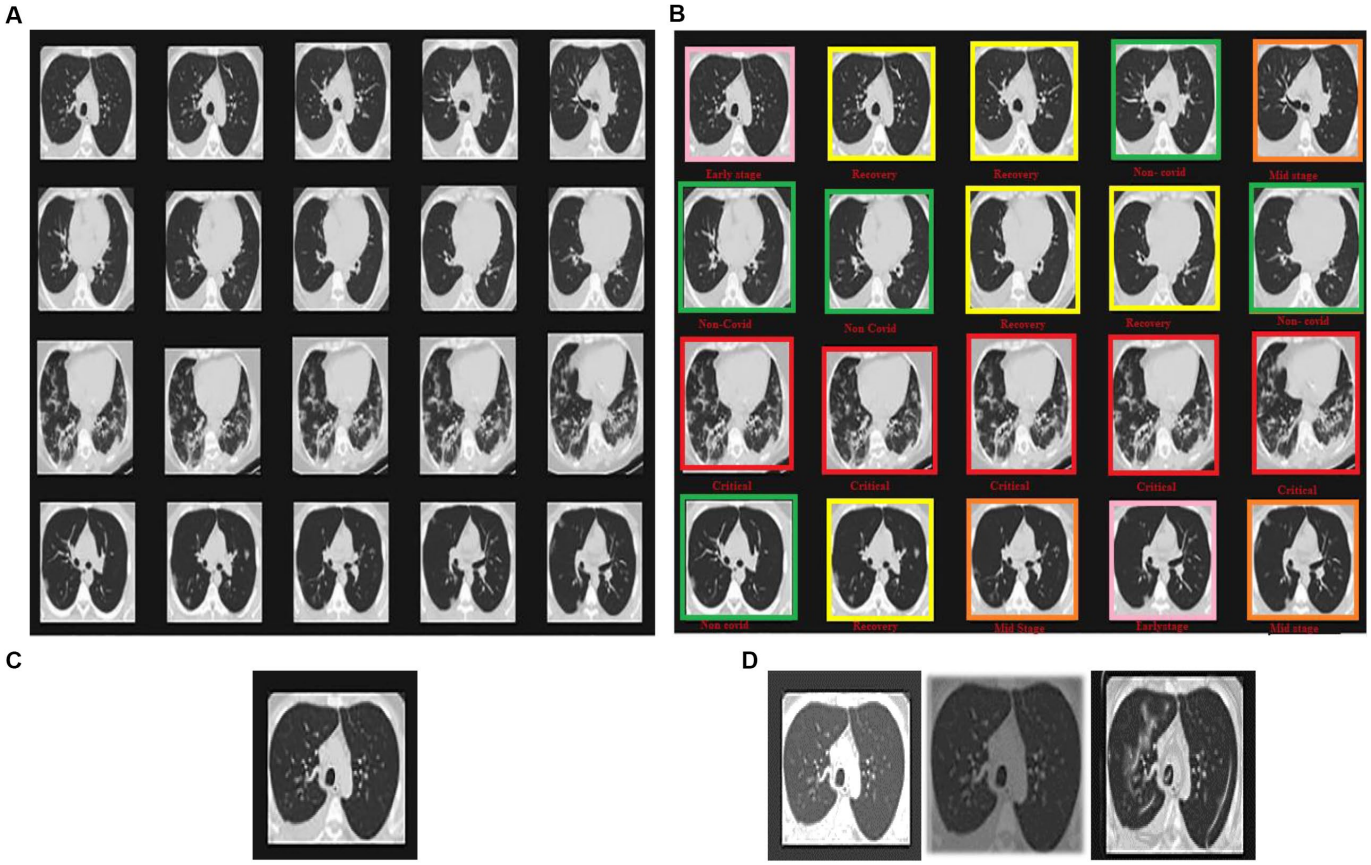
Sample images displaying lung CT images classification in terms of **(A)** 2D axial views, **(B)** COVID-19 severity classes, **(C)** original image, and **(D)** synthetic images of loaded, rescaled, and grayscale.

**Figure 4:**
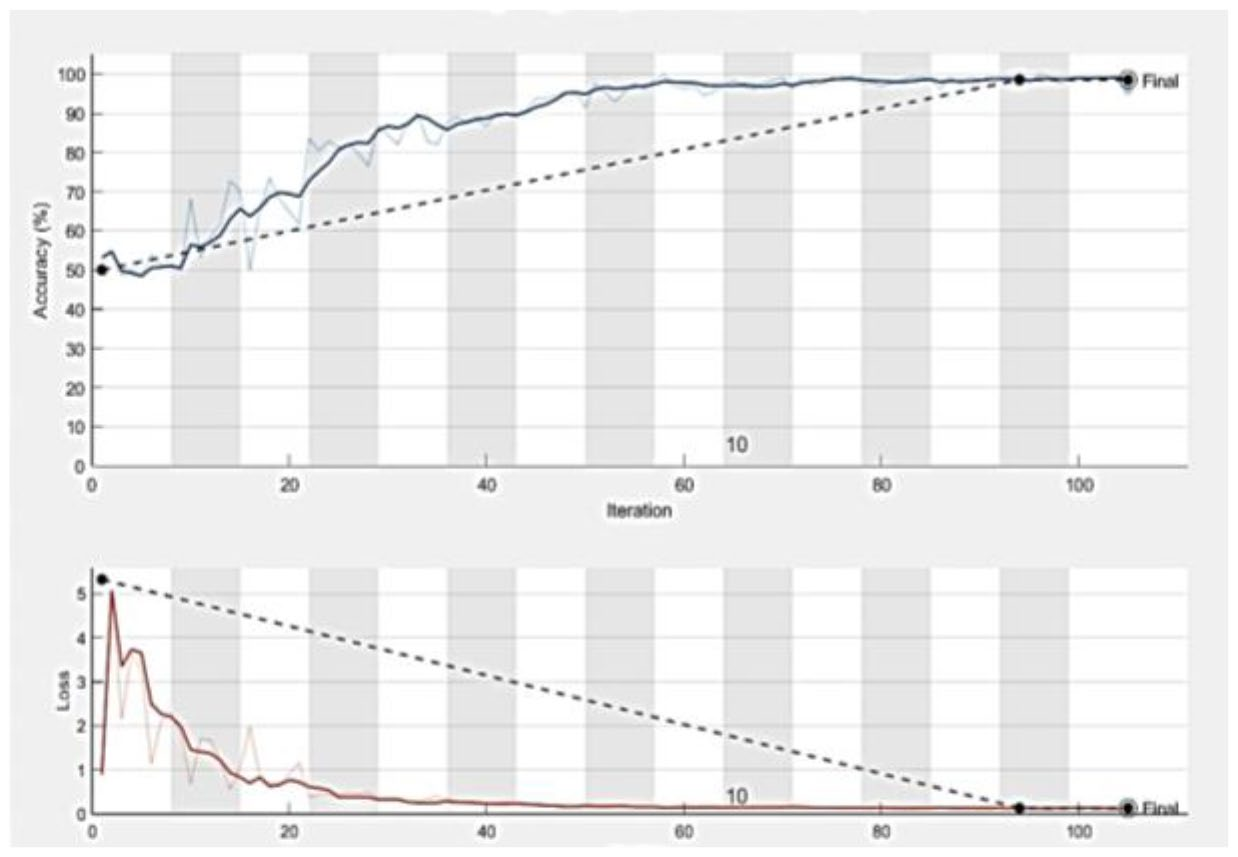
The accuracy and loss curves which tend to be stable after descending, signify that the training process converges.

Statistical research evaluates the effectiveness of the suggested model in identifying defective covid lungs based on a range of performance parameters. Sample lung images are shown in Figure 3. Here, Figure 3A depicts the 2D axial views, and Figure 3B shows covid-19 severity classes. Red signifies instances of life-threatening severity, whereas pink denotes COVID early stages cases, orange denotes patients in the mid-stage of covid, yellow represents patients who have recovered from covid, and green specifies healthy non-covid individuals. Even if the suggested method is successful, it is still feasible to discriminate between covid and non-covid cases using this method. Figures 3C,D demonstrate the original image and the corresponding synthetic images generated using pix2pix GAN. As seen in Figure 4, there exists a considerable rise in loss values when the training begins and a significant reduction in loss values when the training ends. Due to the low number of COVID-19 cases compared to the other two classes (Pneumonia and No-Findings), this dramatic spike and decline are mostly due to this class. It is possible to lessen these high and low points in the training by examining all CT images repeatedly for each epoch of training.

- True negative (TN): Normal lung image is properly identified.
- True positive (TP): The case is correctly distinguished.
- False negative (FN): The case is incorrectly identified.
- False positive (FP): The normal lung image is incorrectly diagnosed.

### 5.2. Accuracy

The percentage of accurate findings based on the total number of data points is called image quality. It is displayed in percentages how accurate and precise the recall is as shown in Equation (39).


(39) $$\mathrm{Accuracy}\;\left(A\right)=\frac{\mathrm{TP}+\mathrm{TN}}{\left(\mathrm{TP}+\mathrm{TN}+\mathrm{FP}+\mathrm{FN}\right)}$$

### 5.3. Specificity

Specificity refers to arbitrary errors to calculate algebraic variability as portrayed in Equation (40).


(40) $$\mathrm{Specificity}=\left[\frac{\mathrm{TN}}{\mathrm{TN}+\mathrm{FP}}\right]$$

### 5.4. Recall

The positive data percentage is appropriately identified as the “true optimistic rate and is estimated as illustrated in Equation (41).


(41) $$\mathrm{Recall}=\left[\frac{\mathrm{TP}}{\left(\mathrm{TP}+\mathrm{FN}\right)}\right]$$

### 5.5. Precision

Precision portrays the conditional probabilities of the actual class and the predicted class. Equation (42) shows the expression for precision.


(42) $$\mathrm{Precision}=\left[\frac{\mathrm{TP}}{\left(\mathrm{TP}+\mathrm{FP}\right)}\right]$$

**Figure 5:**
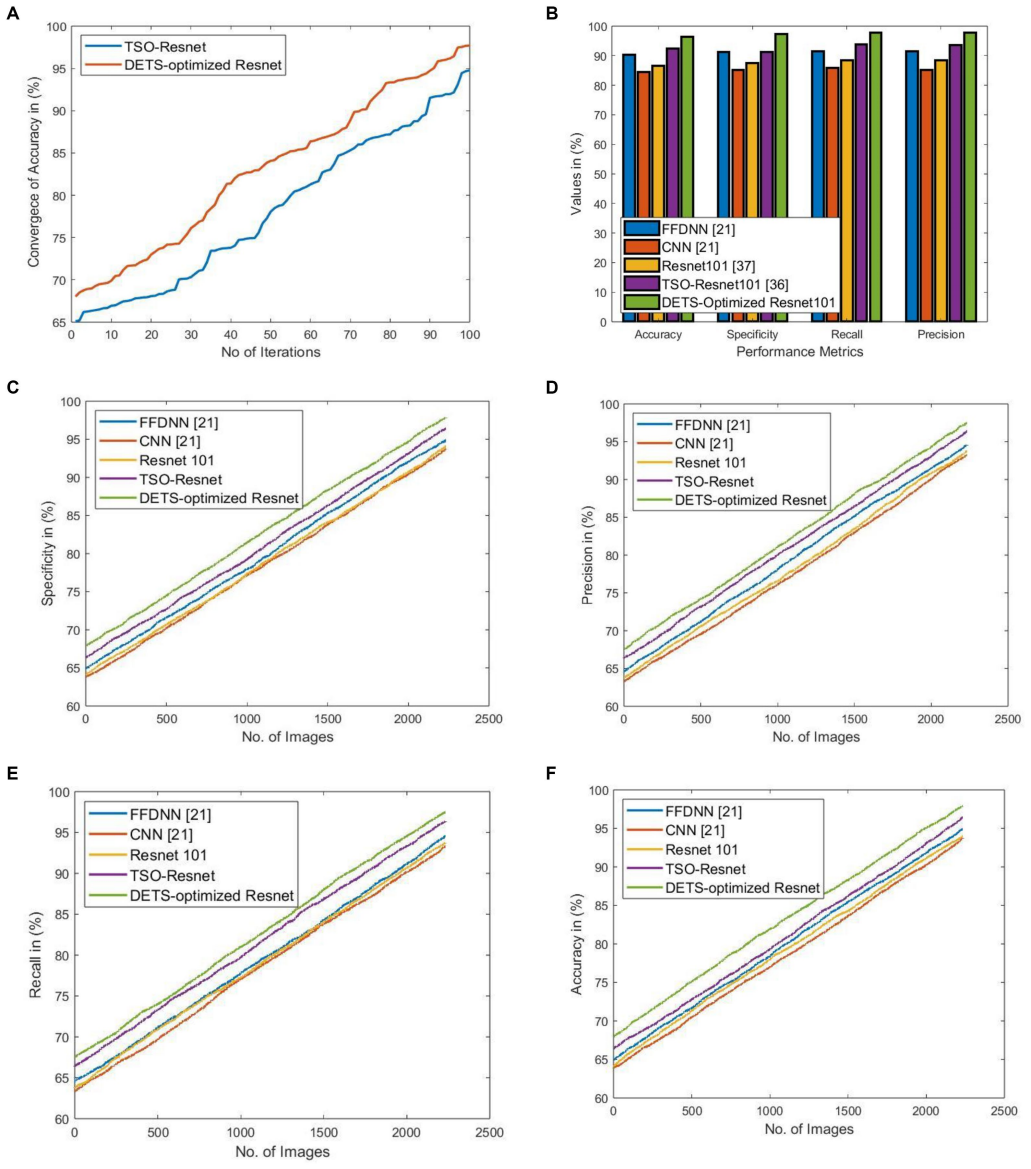
Significance of implemented DETS-optimized Resnet concerning **(A)** convergence of accuracy over TSO-Resnet, **(B)** performance over other methods, **(C)** specificity, **(D)** precision, **(E)** recall, and **(F)** Accuracy.

Figure 5 shows the significance of the proposed DETS-optimized Resnet 101 classifier over traditional methods. Here, Figure 5A shows the convergence of the proposed DETS-optimized Resnet over traditional TSO and accomplished better accuracy which is 2.25% improved than traditional TSO-Resnet.

Figure 5B shows the performance of the proposed DETS-Optimized Resnet 101 classifier over other methods using holdout as 70% of training data and 30% of testing data. For accuracy, the implemented DETS-optimized Resnet 101 model attained 6.32, 12.42, 8.29, and 3.83% better than FFDNN, CNN, Resnet 101, and TSO-based Resnet, respectively. Similarly, for specificity, recall, and precision, the proposed DETS-Optimized Resent 101 classifier performed well than other methods. Besides, Figures 5C–F portray the specificity, precision, recall, and accuracy, respectively, of the proposed DETS-Optimized Resnet over other methods. Herein, 30% of testing data is employed that is 744 images with 378 COVID-19+ and 366 COVID-19 negative people. This investigation demonstrates that the proposed COVID-19 classification system using lung images via DL models performed well and outruns the other models.

**Figure 6:**
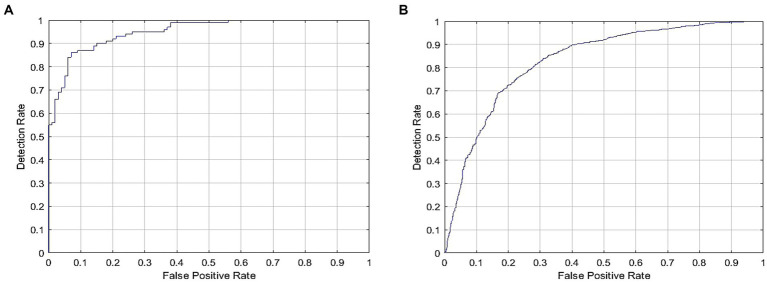
ROC for COVID-19 detection for DL model signifying **(A)** Internal and **(B)** external validation.

Using specific CT images, the model produced 0.95 and 0.89 AUC for internal and external validations, respectively, as shown in Figure 6. Additionally, Table 2 represents the internal and external validations of the proposed DL algorithm where the accuracy is 97.2 and 95.4% for internal and external validations, respectively. Similarly, specificity is 95.5 and 94.62%, precision is 96.7 and 94.25%, and recall is 95.9, and 93.95% for internal and external validations, respectively. Besides, Positive Predictive Value (PPV), and Negative Predictive Value (NPV) are also evaluated to estimate the disease-positive and disease-negative data. Kappa values are estimated to find the interrater reliability among the data which is measured using the proposed DL classification results and clinical results. Also, ROC and Youden index is employed to measure the optimal cutoff between sensitivity and specificity which yielded 0.84 and 0.78 for internal and external validations, respectively. Table 3 summarizes the performance of the proposed DL over skilled radiologists. Around 730 images are given to determine the COVID-19 disease by 2 expert radiologists (R1 and R2). Here, Radiologist 1 (R1) attained 54.89% accuracy whereas Radiologist 2 (R2) achieved 56.21% of accuracy. Also, the specificity precision, recall, F1-score, NPV, and PPV values are low when compared with the proposed DL model. It indicates that the classification of COVID-19 using bare eyes is insignificant when compared with DL algorithms. Table 4 shows the 5-fold cross-validation results of the proposed DETS-ResNet model. Hither, the cross-validation approach is used to distinguish between the training and testing images in the dataset and assess the proposed algorithm’s performance. The entire number of images is divided into five sets. The data from set 1 are utilized for testing and the images from other sets are used for training the first fold. The classification accuracy and other metrics were determined for the first fold taking this into account. For the second fold, the data from set 2 are utilized for testing, while other sets are used for training. The remaining data are utilized as training images for the third fold, fourth fold, and fifth fold metrics, from sets 3, 4, and 5 for testing, respectively. To arrive at the overall results, the metrics acquired for folds 1–5 were averaged. From this assessment, fold 3 attained better results with Adam’s optimizer. The overall performance of the proposed model reached 96.7% accuracy, 96.87% specificity, 97.27% precision, 97% recall, 87.67% NPV, 89.07% PPV, and 96.9% F1-score.

**Table 2:** Performance of proposed DL model.

Measures	Internal	External
AUC	0.95	0.89
Accuracy	97.2%	95.4%
Specificity	95.5%	94.62%
Precision	96.7%	94.23%
Recall	95.9%	93.95%
PPV	86.21%	81.84%
NPV	92.35%	88.56%
Kappa	0.75	0.71
F1-score	96.74%	93.21%
Youden index	0.84	0.78

**Table 3:** Performance measures for proposed DL model over skilled radiologists.

Measures	Internal	External (from input images)	External (from patients)	R1	R2
Accuracy	97.2%	95.4%	90.44%	54.89%	56.21%
Specificity	95.5%	94.62%	89.91%	75.23%	76.41%
Precision	96.7%	94.23%	91.23%	73.22%	73.56%
Recall	95.9%	93.95%	90.22%	71.52%	73.65%
NPV	86.21%	81.84%	91.23%	52.85%	56.21%
PPV	92.35%	88.56%	89.57%	46.21%	48.21%
Kappa	0.75	0.71	0.77	0.25	0.28
F1-score	96.74%	93.21%	92.45%	46.23%	46.84%
Youden index	0.84	0.78	0.75	0.32	0.35

**Table 4:** Performance measures for proposed DL model using 5-fold cross validation.

Measures	1-fold	2-fold	3-fold	4-fold	5-fold	Overall
Accuracy	96.2%	95.7%	97.47%	96.89%	97.25%	96.7%
Specificity	95.01%	94.3%	98.91%	97.8%	98.46%	96.87%
Precision	96.7%	95.3%	98.23%	97.56%	98.56%	97.27%
Recall	95.7%	94.9%	98.22%	97.52%	98.65%	97%
NPV	87.21%	87.8%	91.3%7	85.85%	86.21%	87.67%
PPV	93.35%	83.56%	95.57%	87.7%	85.21%	89.07%
F1-score	95.74%	94.21%	98.45%	97.9%	98.2%	96.9%

### 5.6. Discussion

There has been a pressing need for quicker alternatives that front-line healthcare workers may employ for reliably and swiftly detecting the condition due to the limits of manual tests. In this work, a DL algorithm is proposed for the analysis of sample CT scans. Using the developed DETS-optimized Resnet, which has a 97.2% overall accuracy. More significantly, the developed framework obtained a reasonably high sensitivity of 95.9 and 93.95% on internal and exterior CT images, correspondingly, making it an effective screening approach. Notably, although both COVID-19 and other common viral pneumonia have many radiological features, the proposed model was able to differentiate between them. Therefore, the DETS-optimized Resnet model has the potential as an effective tool for COVID-19 screening during the present worldwide COVID-19 epidemic. There is no segmentation procedure used in this work. In fact, segmentation helps to enhance classification performance. Also, the data used in the experimentation is gathered from a single source which is synthetically augmented. In the future, data from various sources will be gathered and utilized for COVID-19 detection with multi-Modality.

## 6. Conclusion

Using an optimized Resnet 101 model, the chest CT scans were classified between an infected patient and a healthy one. In this investigation, CT scans were used since they reveal the polluted areas of the lungs and were practically universally accessible in hospitals. Other germs, such as pneumonia and flu, become more virulent in frigid temperatures, making it difficult for physicians to determine the severity of an illness. The study’s second goal was to build a collection of exact images using a Pix 2 Pix GAN network based on TL. Stages include early, mid, recovery, critical, and non-covid in the CT pictures of the chest. DETS Optimizer technique and DL-based Resnet 101 approaches were part of the suggested strategy. The accurate pictures were obtained by the application of image reconstruction algorithms. TL-based GAN was applied to a picture that has been randomly generated in both height and width. The suggested model was put to the test in a variety of ways. COVID-19 CT image categorization with five classifications was tested. Based on many assessment factors such as Accuracy, Precision, Recall, and Specificity, the enhanced Resnet 101 was assessed. Four courses yielded the best outcomes. CNN and DNN models were used for the comparison. In all classes, the improved Resnet 101 surpasses CNN and DNN. In the future, image segmentation will be done using a variety of color saturation algorithms to enhance the classification performance. Besides, Hemodialysis patients in Alsaffar (37), can use the proposed model instead of statistical data. Image datasets can be used with the effectiveness of Deep Learning algorithms to identify more precisely. Moreover, it can reduce the highly time-consuming process of manual diagnosis. For this process, the proposed optimized Resnet 101 can be employed to reduce the manual workload and can help in early detection.

## Data availability statement

The datasets presented in this study can be found in online repositories. The names of the repository/repositories and accession number(s) can be found at: https://www.kaggle.com/datasets/plameneduardo/sarscov2-ctscan-datase0074.

## Ethics statement

Ethical approval was not required for the study involving humans in accordance with the local legislation and institutional requirements. Written informed consent to participate in this study was not required from the participants or the participants’ legal guardians/next of kin in accordance with the national legislation and the institutional requirements.

## Author contributions

All authors listed have made a substantial, direct, and intellectual contribution to the work and approved it for publication.

## Conflict of interest

The authors declare that the research was conducted in the absence of any commercial or financial relationships that could be construed as a potential conflict of interest.

## Publisher’s note

All claims expressed in this article are solely those of the authors and do not necessarily represent those of their affiliated organizations, or those of the publisher, the editors and the reviewers. Any product that may be evaluated in this article, or claim that may be made by its manufacturer, is not guaranteed or endorsed by the publisher.
